# Extragingival Pyogenic Granuloma: an Unusual Clinical Presentation

**Published:** 2015-09

**Authors:** Suresh K. Sachdeva

**Affiliations:** a Dept. of Oral Medicine and Radiology, Surendera Dental College and Research Institute, Sriganganagar-335001, Rajasthan, India.

**Keywords:** Buccal mucosa, Extra gingival, Female, Pyogenic granuloma

## Abstract

Pyogenic granuloma is thought to represent an exuberant tissue reaction to local irritation. It occurs in second decade of life in young females. Clinically, oral pyogenic granuloma is a smooth or lobulated exophytic growth, pedunculated or sessile, which usually bleeds on provocation. Oral pyogenic granuloma preferentially affects the gingiva. On rare occasion, it can be found extragingivally on lips, tongue, buccal mucosa, and palate which may mimic more serious pathological conditions such as malignancies. This article reports an unusual case of extra gingival pyogenic granuloma occurring on the right buccal mucosa in a female patient and discusses the features that distinguish this lesion from other similar oral mucosal lesions.

## Introduction

Pyogenic granuloma is a benign, soft tissue, non-neoplastic lesion of the oral cavity, thought to represent a reactive exuberant tissue-reaction to local irritation or trauma.(1) The term pyogenic granuloma is a misnomer as it does not produce pus and does not show granulomatous changes, microscopically.(2) Clinically, pyogenic granuloma usually presents as solitary exophytic growth, which may be sessile or pedunculated with smooth or lobulated surface. The color of the growth is red in younger lesions and with maturity; it becomes more pink and fibrous as the vascularity decreases. It predominantly occurs in second decade of life in young females (female: male=2:1). In general, pyogenic granuloma is asymptomatic but sometimes it can be painful, especially in an area of continuous trauma.(3) In the oral cavity, the most common site of involvement is the gingiva (75%), maxilla being more involved than mandible. In rare instances, it may occur extragingivally on the lips, tongue, buccal mucosa, and palate.(4) The purpose of this article is to report a rare case of extra gingival pyogenic granuloma occurring on the right buccal mucosa in a female and to distinguish this lesion from other similar lesions of the buccal mucosa, with special emphasis on the diagnosis and treatment of this condition.

## Case Report

**Figure 1:**
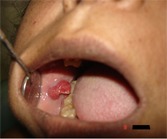
Intra-oral bright red growth on the right buccal mucosa at the level of occlusal line.

A 45-year-old female patient reported with the chief complaint of growth on the inner aspect of right cheek with one-year duration. History of presenting illness revealed that the growth was gradual in onset, initially small in size now increased to attain the present size. There was also a history of occasional bleeding from the growth during chewing. The patient’s medical history and family was insignificant. Extra oral examination showed no swelling or facial asymmetry. Intraoral examination revealed a solitary exophytic, pedunculated growth on the right side of buccal mucosa at the level of occlusal plane (Figure 1).

The exophytic growth appears erythematous with grayish white borders with lobulated surface. The growth measured of about 2cm×1cm in diameter, which was soft to firm in consistency and bled on provocation. The cusp of the right upper premolars had sharp buccal edge corresponding to about the center of the swelling. There was no evidence of pus discharge from the lesion. Regional lymph nodes were not palpable. Based on the history and clinical appearance of the lesion, provisional diagnosis of benign exophytic growth of right buccal mucosa was considered. And the differential diagnosis included traumatic fibroma, pyogenic granuloma, and capillary hemangioma. The lesion was excised under local anesthesia and sent for histopathological examination. 

**Figure 2:**
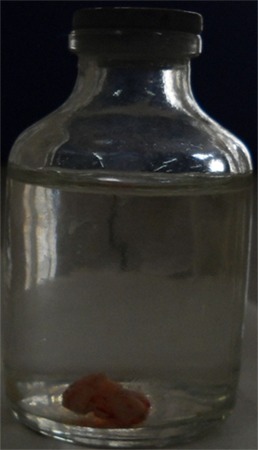
Excised specimen in the formalin bottle.

**Figure 3:**
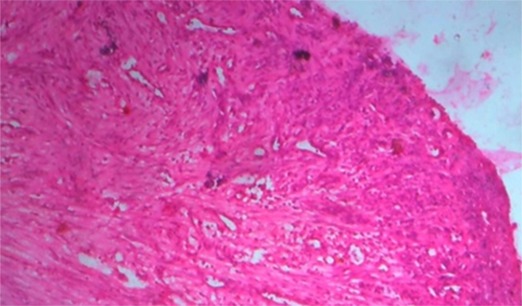
Histopathological photomicrograph showing features of pyogenic granuloma (H & E, x40)

Figure 2 shows the macroscopic appearance of the excised specimen in the formalin bottle. Histopathologically, hematoxylin-eosin (H&E) stained section confirmed the clinical diagnosis of pyogenic granuloma (Figure 3).

**Figure 4:**
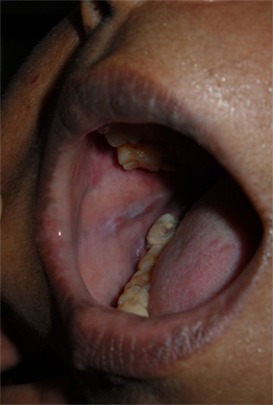
Post-operative photograph of the same patient.

The wound healing was completed and no recurrence was noted on 3-year follow up period (Figure 4).

## Discussion

Hullihen (1844) was first to define pyogenic granuloma in the English literature. In 1897, pyogenic granuloma in man was designated as botryomycosis hominis. Hartzell (1904) is credited with giving the current term of pyogenic granuloma or granuloma pyogenicum. It was also called as Crocker and Hartzell's disease. Angelopoulos histologically defined it as hemangiomatous granuloma due to the presence of frequent blood vessels and the inflammatory nature of the lesion.(5)

This lesion is formed as a result of exaggerated localized tissue reaction to a trauma or any irritation like calculus, poor oral hygiene, nonspecific infection, over hanging restorations, cheek biting, previous dental extractions, exfoliating primary teeth, bone spicules, root remnants, tooth brush trauma.(2) Pyogenic granuloma is predominantly seen in second decade of life in young adult females, possibly because of vascular effects of female hormones.(3) It occurs in 1% of pregnant women, although, Muench MG *et al.*(6) reported a rare case of pyogenic granuloma in a 6-day old patient associated with natal tooth in anterior mandibular region.

The incidence of pyogenic granuloma is 26.8 to 32% of all reactive lesions.(7) In the oral cavity, pyogenic granuloma has site preference for the gingiva (75% of the cases) specifically, interdental papillae. On rare occasion, it can be found extragingivally in the area of frequent trauma, such as lower lip, tongue, palate and buccal mucosa.(4) In the present case, it arises extragingivally from right buccal mucosa. The constant trauma from adjacent sharp cusp tip could be the reason for this location.

Clinically, the appearance of pyogenic granuloma is red/pink to purple, and can be smooth or lobulated mass which may be pedunculated or sessile. Younger lesions are more likely to be red because of the high number of blood vessels. Older lesions begin to change into a pink color. Pyogenic granuloma grows in size up to several centimeters in size but is usually less than 2.5cm.(3) It can be painful, especially if located in an area of the body where it is constantly disturbed. Pyogenic granuloma can grow rapidly and will often bleed profusely with little or no trauma.(8) In this case, the size of the lesion was 2cm×1cm. Pyogenic granuloma can be diagnosed clinically with considerable accuracy, radiographic and histopathological investigations aid in confirming the diagnosis and treatment. Radiographs are advised to rule out bony destructions suggestive of malignancy or to identify a foreign body. Moreover, the radiographs help in determining the alveolar bone erosion leading to mobility and tooth loss. The differential diagnosis of pyogenic granuloma includes clinically similar conditions such as traumatic fibroma, parulis, post-extraction granuloma, peripheral giant cell granuloma, peripheral ossifying fibroma, peripheral odontogenic fibroma, hyperplastic gingival inflammation, Kaposi’s sarcoma, amelanotic melanoma, bacillary angiomatosis, angiosarcoma, metastatic cancer, non Hodgkin’s lymphoma, and hemangioma.(9) Therefore, all clinically suspected pyogenic granuloma must be histopathologically examined to rule out other benign and malignant conditions. Differentiation is done on clinical and histological features which also help in adequate treatment and good prognosis. The histopathological picture of the extragingival pyogenic granuloma is similar to the gingival pyogenic granuloma. Histopathologically, it consists of many dilated blood vessels in a loose edematous connective tissue stroma. Sometimes, these vessels are organized in lobular aggregates and called as lobular capillary hemangioma.(3)

Management of pyogenic granuloma is conservative surgical excision, after thorough oral prophylaxis. If the lesion is small, painless and free of bleeding, oral prophylaxis and removal of causative irritants is advised. If the lesion is of large size, a thorough oral prophylaxis followed by surgical excision using gingivectomy or flap surgery procedures is done.(10) Other treatment protocols have also been proposed such as cryosurgery which is safe, easy and inexpensive; and also Nd: YAG and CO_2_ and flash lamp pulsed dye lasers. Lasers have advantage of minimum pain and invasiveness and the lack of need for suturing or packing.(11) Moon *et al.*(12) reported that sodium tetradecyl sulfate sclerotherapy successfully cleared the lesions in most patients without major complications. Parisi *et al.*(8) used a series of intralesional corticosteroid injections for the treatment of recurrent pyogenic granuloma. Treatment during pregnancy ranges from preventive measures such as careful oral hygiene, removal of dental plaque, and use of a soft toothbrush. In some cases, shrinkage of the lesion after pregnancy may make surgical treatment unnecessary However, if necessary treatment can be completed in the second trimester with follow up.(13) After excision, recurrence occurs in up to 16% of the cases. Recurrence is believed to result from incomplete excision, failure to remove etiologic factors, or re-injury of the area. Recurrence of extra gingival pyogenic granuloma is uncommon.

## Conclusion

As the occurrence of pyogenic granuloma in extra gingival sites is unusual, this case report emphasizes the importance of the correct diagnosis of this lesion and differentiating this from other benign and malignant oral mucosal lesion which have similar characteristics. Subsequently, the oral physicians should be aware of occurrence of these type of lesions on uncommon sites and there proper management. 
